# Analysis of viral load in different specimen types and serum antibody levels of COVID-19 patients

**DOI:** 10.1186/s12967-020-02693-2

**Published:** 2021-01-07

**Authors:** Ling Li, Chianru Tan, Jia Zeng, Chen Luo, Shi Hu, Yanke Peng, Wenjuan Li, Zhixiong Xie, Yueming Ling, Xuejun Zhang, E. Deng, Haixia Xu, Jue Wang, Yudi Xie, Yaling Zhou, Wei Zhang, Yong Guo, Zhong Liu

**Affiliations:** 1 Institute of Blood Transfusion, Chinese Academy of Medical Sciences and Peking Union Medical College, Chengdu, 610052 Sichuan People’s Republic of China; 2 Key Laboratory of Transfusion Adverse Reactions, Chinese Academy of Medical Sciences, Chengdu, 610052 Sichuan People’s Republic of China; 3 Department of Biomedical Engineering, School of Medicine, Tsinghua University, 30 Shuangqing Road, Beijing, 100084 People’s Republic of China; 4 Department of Aviation Disease, Naval Medical Center of PLA, Second Military Medical University, Shanghai, 200052 People’s Republic of China; 5 The Maternal and Child Health Hospital of Hubei Province, Guanggu District, Wuhan, 430070 Hubei People’s Republic of China; 6 Department of Biophysics, College of Basic Medical Sciences, Second Military Medical University, Shanghai, 200433 People’s Republic of China; 7 Anhui Medical University, Hefei, 230032 People’s Republic of China; 8 Department of Clinical Laboratory Science of NO. 909 Hospital of PLA Joint Support Force, Zhangzhou, 363000 People’s Republic of China; 9 Department of Clinical Laboratory Science of NO. 910 Hospital of PLA Joint Support Force, Quanzhou, 362000 People’s Republic of China; 10 Department of Respiratory and Critical Care Medicine, First Affiliated Hospital, Second Military Medical University, 168# Changhai Rd, Shanghai, 200433 People’s Republic of China

**Keywords:** COVID-19, Viral load, Droplet digital PCR, Nasopharyngeal swab

## Abstract

**Background:**

COVID-19 has caused a global pandemic and the death toll is increasing. However, there is no definitive information regarding the type of clinical specimens that is the best for SARS-CoV-2 detection, the antibody levels in patients with different duration of disease, and the relationship between antibody level and viral load.

**Methods:**

Nasopharyngeal swabs, anal swabs, saliva, blood, and urine specimens were collected from patients with a course of disease ranging from 7 to 69 days. Viral load in different specimen types was measured using droplet digital PCR (ddPCR). Meanwhile, anti-nucleocapsid protein (anti-N) IgM and IgG antibodies and anti-spike protein receptor-binding domain (anti-S-RBD) IgG antibody in all serum samples were tested using ELISA.

**Results:**

The positive detection rate in nasopharyngeal swab was the highest (54.05%), followed by anal swab (24.32%), and the positive detection rate in saliva, blood, and urine was 16.22%, 10.81%, and 5.41%, respectively. However, some patients with negative nasopharyngeal swabs had other specimens tested positive. There was no significant correlation between antibody level and days after symptoms onset or viral load.

**Conclusions:**

Other specimens could be positive in patients with negative nasopharyngeal swabs, suggesting that for patients in the recovery period, specimens other than nasopharyngeal swabs should also be tested to avoid false negative results, and anal swabs are recommended. The antibody level had no correlation with days after symptoms onset or the viral load of nasopharyngeal swabs, suggesting that the antibody level may also be affected by other factors.

## Background

The recent emergence of the severe acute respiratory syndrome coronavirus 2 (SARS-CoV-2) poses a serious threat to human health. SARS-CoV-2 can cause asymptomatic infections, mild self-limiting respiratory diseases, and severe progressive pneumonia (resulting in shock, acute respiratory distress syndrome (ARDS), acute heart injury, acute kidney injury, and death) [1, 2]. World Health Organization (WHO) named the disease caused by SARS-CoV-2 infection the coronavirus disease 2019 (COVID-19), which has rapidly expanded across the globe. A rapid and reliable diagnosis of COVID-19 is critical for control of this pandemic.

Quantitative reverse transcription polymerase chain reaction (RT-qPCR) is the main diagnostic method to identify patients with COVID-19. Proper specimen collection is important for the diagnosis [3]. Studies have found that SARS-CoV-2 nucleic acid could be detected in nasopharyngeal swabs, sputum, saliva, blood, urine, and anal swabs/feces of COVID-19 patients [4], and the positive detection rate of sputum was the highest, followed by nasopharyngeal swabs [5]. However, not all patients are able to produce sputum, especially elderly patients and patients with endotracheal intubation, which makes it difficult to extract sputum. In addition, the high viscosity of sputum makes it difficult to extract nucleic acids. Therefore, most specimens collected at present are nasopharyngeal swabs. However, poor quality of nasopharyngeal swabs collection could contribute to false-negative results, and it has been reported that the rectal/anal swabs from some patients who in the recovery stage were persistently tested positive after the nasopharyngeal testing was negative [6, 7]. Thus, further research is needed to establish which specimen types are most suitable for SARS-CoV-2 nucleic acid detection.

In addition to nucleic acid detection, virus-specific antibody detection is of great significance for auxiliary diagnosis, differential diagnosis, and monitoring the disease progression and treatment effect. Researchers have studied antibody kinetics to determine the seroconversion rate and median seroconversion time of patients with COVID-19 [8] as well as the relationship between disease severity and the antibody level [9]. However, the antibody levels in patients with different duration of disease and the relationship between antibody level and viral load remained unclear.

In this study, we collected nasopharyngeal swabs, anal swabs, saliva, blood, and urine specimens of COVID-19 patients with a disease course of 7–69 days, and used droplet digital (ddPCR) to detect SARS-CoV-2 in these different specimens. We also measured the anti-nucleocapsid protein (anti-N) IgM and IgG levels in the serum of these patients and the titer of anti-spike protein receptor-binding domain (anti-S-RBD) IgG to study the correlation between antibody production and viral load in COVID-19 patients.

## Methods

### Ethics statement

The study was approved by the Ethics Committee of the Institute of Blood Transfusion, Chinese Academy of Medical Sciences & Peking Union Medical College. Written informed consent was obtained from each study participant.

### Participants

A total of 185 samples from 37 patients with COVID-19 were collected in Maternal and Child Health Care Hospital of Hubei Province (Guanggu District) between March 17 and March 24, 2020. Nasopharyngeal swab, anal swab, saliva, blood, and urine were collected from each patient. Written informed consent was obtained from all patients before the study.

### Clinical classification of COVID-19

The diagnosis was based on the *Diagnosis and Treatment Protocol for COVID-19* (trial version 7) established by the National Health Commission of the People’s Republic of China [10]. The clinical classification of COVID-19 was as follows: (1) Mild: mild clinical symptoms with no sign of pneumonia on imaging. (2) Moderate: showing fever and respiratory symptoms with radiological findings of pneumonia. (3) Severe: adult cases meeting any of the following criteria: (a) respiratory distress (≥ 30 breaths/min); (b) oxygen saturation ≤ 93% at rest; (c) arterial partial pressure of oxygen (PaO_2_)/fraction of inspired oxygen (FiO_2_) ≤ 300 mmHg (1 mmHg = 0.133 kPa); (d) cases with chest imaging that shows obvious lesion progression within 24–48 h > 50%. (4) Critical: meeting any of the following criteria: (a) respiratory failure and mechanical ventilation is required; (b) shock; (c) with other organ failure that requires ICU care.

### Specimen collection and transportation

Nasopharyngeal swab, anal swab, saliva, blood, and urine were collected and stored at 4 °C until use. Sampling methods were as follows:

1. Nasopharyngeal swab: The patient was instructed to rinse his/her mouth with water, and then a swab was inserted through the nostril parallel to the palate. The swab was left in the nasopharynx for 15 s and gently rotated three times. The swab was then withdrawn and placed into a collection tube.
2. Anal swab: A sampling swab was soaked in normal saline, and then inserted 2–3 cm deep into the anus. The swab was used to wipe the fold around the anus or gently rub against the anal opening. The swab was withdrawn and placed into a tube containing normal saline.
3. Saliva: In the morning, the patient was instructed to rinse his/her mouth with water and then rest for 10 min. Then, the initial saliva was discarded, and the patient spit at least 2 ml of saliva into the collection tube. If the patient was not able to produce a sufficient amount of saliva, oral and tongue exercise was used to promote saliva secretion. The cap was tightened after collection and the tube was turned upside down five times.

4. Blood: A vacuum negative-pressure blood collection tube was used to collect 5 ml of blood each in a heparin anticoagulation tube and an EDTA anticoagulation tube. The blood specimens were left at room temperature for 30 min.
5. Urine: Morning urine was collected. The initial urine was discarded and the middle urine was collected. To collect urine from a urine catheter, the urinary catheter was clamped and the sampling site was disinfected with alcohol. The catheter was punctured with a sterile syringe to draw urine into a collection tube.

### RNA extraction and ddPCR detection of viral load in the different specimens

Samples were collected and soaked in 1000 μl PBS buffer. Viral RNA was extracted within 2 h according to the manufacturer’s instructions using the MagPure Viral Nucleic Acid Duo Kit (Magen, Guangzhou, China).

The 185 samples were tested using ddPCR. The ddPCR was performed using a SARS-CoV-2 nucleic acid detection kit (ddPCR) (TargetingOne, Beijing, China) and a TD-1™ Droplet Digital™ PCR system (TargetingOne, Beijing, China, licensed in China, registration number: 20170025, 20192220517) following the manufacturer’s instructions.

### Detection of anti-N IgM and IgG antibodies

Anti-N IgM and IgG antibodies in all serum samples except for one patient were tested by the enzyme-linked immunosorbent assay (ELISA) according to the manufacturer’s instructions. The anti-N IgM and IgG antibody detection kits were purchased from domestic pharmaceutical group Inc (China).

### Titer of anti-S-RBD IgG antibody

Anti-S-RBD IgG antibody in all serum samples except for one patient were tested using the ELISA assay as previously described [11]. Results were reported as the ratio of a sample’s OD value (S) to the cut-off value (CO), i.e., the S/CO value. Samples were diluted serially and tested by the ELISA assay. Titers were reported as the highest dilution when the ELISA assay was still positive, ranging between 1:160 and 1:2560 (ELISA endpoint dilution titers).

## Results

### Basic characteristics of the patients

The median age of the 37 patients was 57 years (range 30–94 years), including 26 women and 11 men. Most of the patients had moderate cases, with four patients with severe or critical cases. Table 1 shows the baseline characteristics and routine blood results of the 37 patients with COVID-19.

**Table 1:** Baseline characteristics of COVID-19 patients

Patient	Sex^a^	Age (years)	Time between symptom onset and sampling (days)	Severity	Underlying diseases	White-cell count (× 1000/μl)	Lymphocyte count (× 1000/μl)	Platelet count (× 1000/μl)	CRP (mg/l)	IL-6 (pg/ml)	PT (s)	APTT (s)	TT (s)	FIB (g/L)	D-dimer (μg/ml)	Urea nitrogen (mmol/l)	Serum creatinine (μmol/l)
GG01	F	42	53	Moderate	Yes	7	2.08	289	2.78	1.5	11.7	31.8	14.2	3.18	0.18	3.45	48.9
GG02	F	94	55	Moderate	No	3.3	1.08	354	2.66	91.11	11.4	28.6	17.7	2.17	4.17	5.96	43.6
GG03	M	36	7	Moderate	Yes	8.5	2.15	225	7.37	2.5	12	32.7	12.9	4.79	0.59	3.85	75.1
GG04	F	52	57	Moderate	Yes	9.5	1.32	253	18.22	–	–	–	–	–	–	–	–
GG05	F	73	56	Moderate	No	4.8	1.35	211	1.68	1.5	11.7	37.7	15.1	3.55	1.05	5.5	58.3
GG06	F	55	32	Moderate	No	4.9	1.69	175	0.21	–	10.5	32.3	17.2	2.6	0.23	5.59	85.3
GG07	F	65	34	Moderate	No	–	–	–	–	–	–	–	–	–	–	–	–
GG08	F	85	44	Moderate	Yes	4.1	1.13	200	1	1.5	11.7	35.9	17	2.31	0.58	3.57	57.1
GG09	M	57	16	Moderate	No	5.1	3.05	213	0.26	1.5	11.3	31.3	13.8	2.58	0.17	5.27	58.1
GG10	M	36	50	Moderate	No	5.7	1.42	239	2.84	–	12	32.7	14.6	3.1	0.27	3.44	53.5
GG11	F	37	48	Moderate	No	–	–	–	–	–	–	–	–	–	–	–	–
GG12	M	71	30	Severe	Yes	5.2	0.93	72	1.51	29.51	–	–	–	–	–	3.75	82.9
GG13	M	50	40	Moderate	No	7.7	1.95	277	0.65	–	–	–	–	–	–	4.19	77.5
GG14	M	76	46	Moderate	Yes	–	–	–	–	42.82	–	–	–	–	–	–	–
GG15	F	30	55	Moderate	No	3.2	0.97	140	0.57	–	–	–	–	–	–	–	–
GG16	F	43	20	Moderate	No	8.1	1.87	186	0.72	1.5	10.2	26.3	15.3	3.1	0.25	3.88	62
GG17	F	41	47	Moderate	No	5	3.21	284	1.75	–	8.7	28.8	14.7	3.14	0.22	4.64	58.9
GG18	M	70	36	Moderate	Yes	–	–	–	–	–	–	–	–	–	–	–	–
GG19	F	51	38	Moderate	No	5	1.77	281	0.5	1.5	–	–	–	–	–	–	–
GG20	F	80	42	Moderate	No	5.1	0.87	181	8.37	2.79	–	–	–	–	–	4.23	63.4
GG21	F	69	58	Moderate	No	5.9	1.52	141	3.67	–	11.6	34.8	15	2.27	0.66	5.63	60.6
GG22	F	62	39	Moderate	Yes	4.9	0.9	167	0.04	–	–	–	–	–	–	5.47	56.2
GG23	F	37	69	Moderate	No	4.7	1.69	231	0.08	1.5	11.7	29.9	14.5	2.86	0.17	3.73	50.2
GG24	F	59	52	Moderate	No	–	–	–	–	–	–	–	–	–	–	–	–
GG25	F	87	26	Severe	Yes	9.8	1.37	125	0.41	14	10.8	26.8	20.6	1.84	1.62	6.44	49.5
GG26	F	52	30	Moderate	No	3.4	1.47	343	0.08	1.5	11.9	30.4	16.8	2.38	0.16	4.49	63.3
GG27	M	55	46	Moderate	No	4.6	1.46	346	2.05	1.5	12.6	30.9	15.1	3.1	0.83	3.64	68.3
GG28	F	65	41	Moderate	No	4.1	1.36	193	1.72	–	–	–	–	–	–	7.12	53.7
GG29	F	56	57	Moderate	No	–	–	–	–	–	–	–	–	–	–	–	–
GG30	M	74	58	Moderate	Yes	6.6	1.64	322	1.03	1.5	11.5	33.3	14.5	3.14	0.15	4.11	69.2
GG31	F	86	60	Critical	Yes	11.3	1.27	180	4.9	2.06	11.7	29.6	14.3	2.93	1.6	6.18	65.2
GG32	F	46	67	Moderate	No	3.4	1.19	189	1.05	1.5	11.3	33.6	15.2	2.6	0.24	4.63	51.8
GG33	M	67	26	Critical	No	18.1	2.1	23	9.23	1554.8	18	39.5	24.9	5.64	17.67	13.17	59.5
GG34	F	80	50	Moderate	Yes	3.8	1.18	192	6.03	16.3	11.7	32.5	16.5	2.96	5.73	4.04	54.9
GG35	F	60	53	Moderate	Yes	4.2	0.9	161	0.9	1.5	11.6	25.5	16.3	2.15	0.42	5.21	50.5
GG36	F	53	42	Moderate	No	6.1	1.56	226	1.06	1.5	10.5	31.2	13.4	3.03	0.18	4.26	51.9
GG37	F	77	34	Moderate	No	6.1	1.07	25	0.78	< 1.5	11.2	27.9	15.5	2.9	0.61	3.9	50.5

^a^F = female, M = male

### Positive detection rates and viral load of samples from different sites

**Fig. 1:**
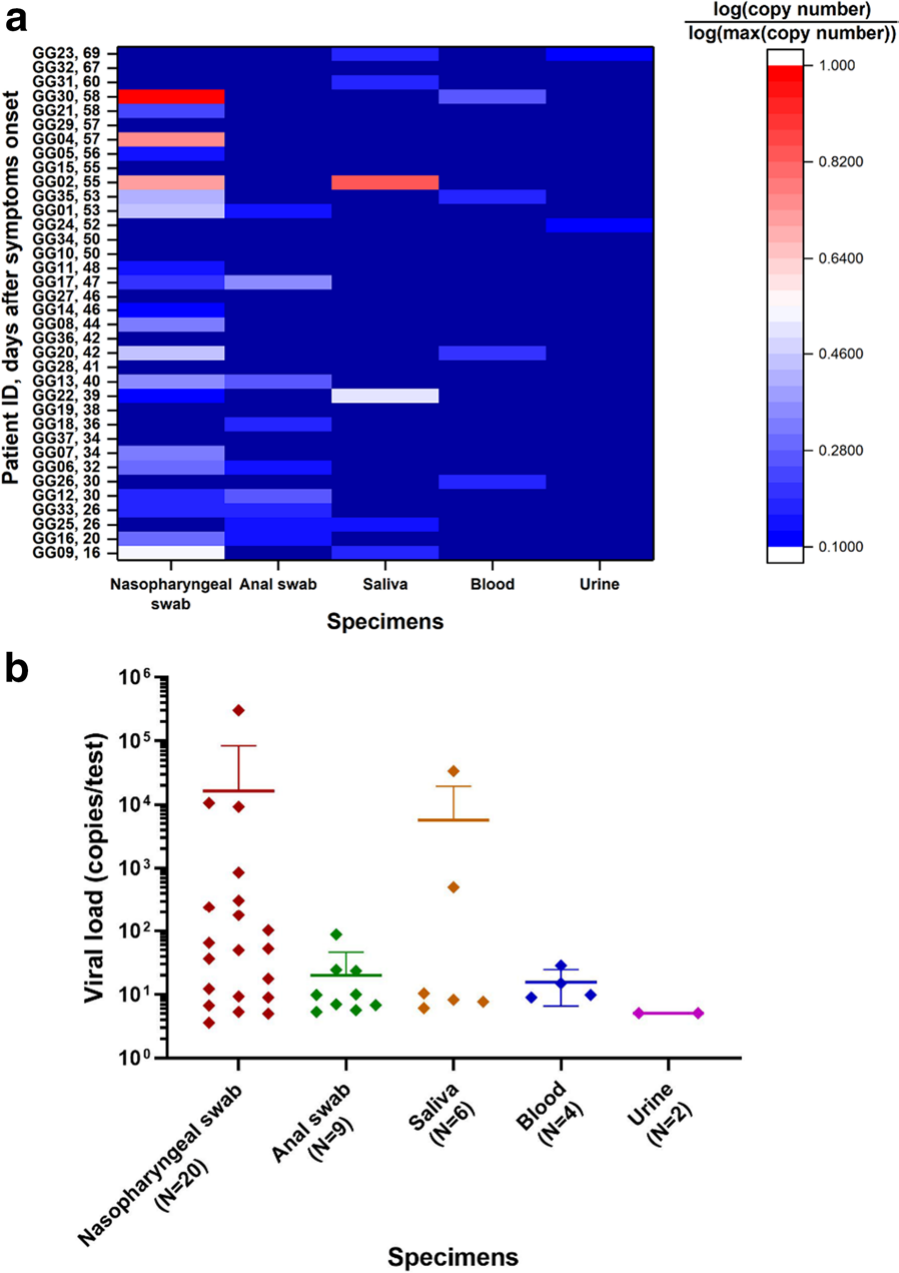
Viral nucleic acid test results. **a** Nucleic acid test results of different specimens from different patients. The x-axis is the specimen, and the y-axis is the patients and days after symptoms onset. The y-axis shows the number of days of illness in descending order from top to bottom. Each cell is colored by viral load. **b** Viral load of different specimens from different patients. The mean copy number and standard deviation are shown in the figure

As shown in Fig. 1a, b, the nasopharyngeal swab had the highest nucleic acid positivity rate at 54.05% (20/37), followed by the anal swab at 24.32% (9/37), and the saliva, blood, and urine were at 16.22% (6/37), 10.81% (4/37), and 5.41% (2/37), respectively. As shown in Fig. 1a, two patients had positive anal swabs but negative nasopharyngeal swabs, and one patient had only a positive anal swab among all specimens. Three patients had positive saliva specimens but negative nasopharyngeal swabs, with one of them having no other positive specimens. One patient was tested positive only in the blood. Of the two patients with positive urine nucleic acid, one patient had no other positive specimens and the other was also positive for the saliva and urine specimens. As shown in Fig. 1b, the mean viral loads of nasopharyngeal swabs, anal swabs, saliva, blood, and urine specimens were 16,224 ± 67,507, 20 ± 26, 5677 ± 13,647, 16 ± 9, and 5.1 ± 0 copies/test, respectively.

### Relationship between antibody levels and days after symptoms onset

**Fig. 2:**
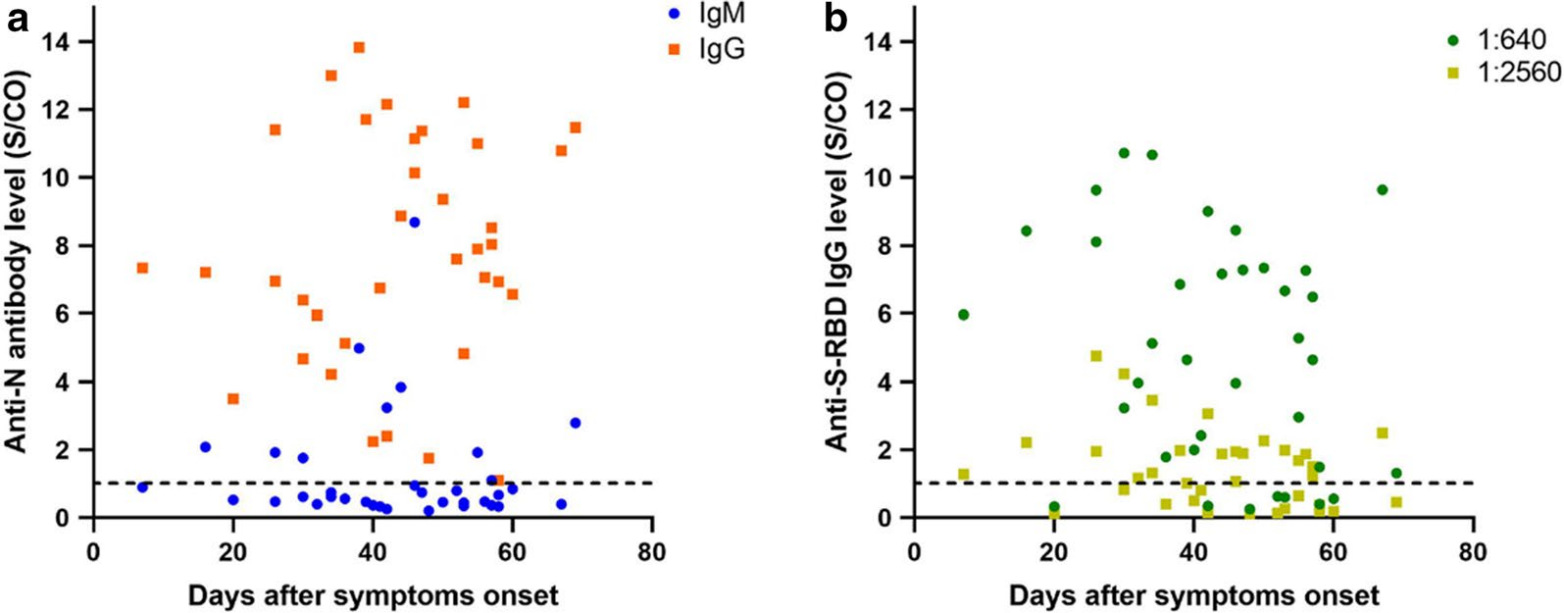
Relationship between **a** anti-N IgG/IgM antibody levels or **b** anti-S-RBD IgG level and days after symptoms onset. The dashed line shows the threshold (S/CO ratio ≥ 1)

Figure 2a demonstrates the relationship between anti-N IgM/IgG antibody levels and days after symptoms onset. Most patients had low levels of IgM antibodies, with only 9 patients (25%) having positive anti-N IgM (S/CO ratio ≥ 1). All patients had positive anti-N IgG (100%), but no significant correlation was observed between anti-N IgM/IgG levels and days after symptoms onset. Figure 2b shows the relationship between anti-S-RBD IgG antibody level and days after symptoms onset; 7 patients (19%) had an anti-S-RBD IgG titer of less than 1:640, and again, no significant correlation between anti-S-RBD IgG level and days after symptoms onset was observed.

### Relationship between antibody levels and viral load of nasopharyngeal swabs

**Fig. 3:**
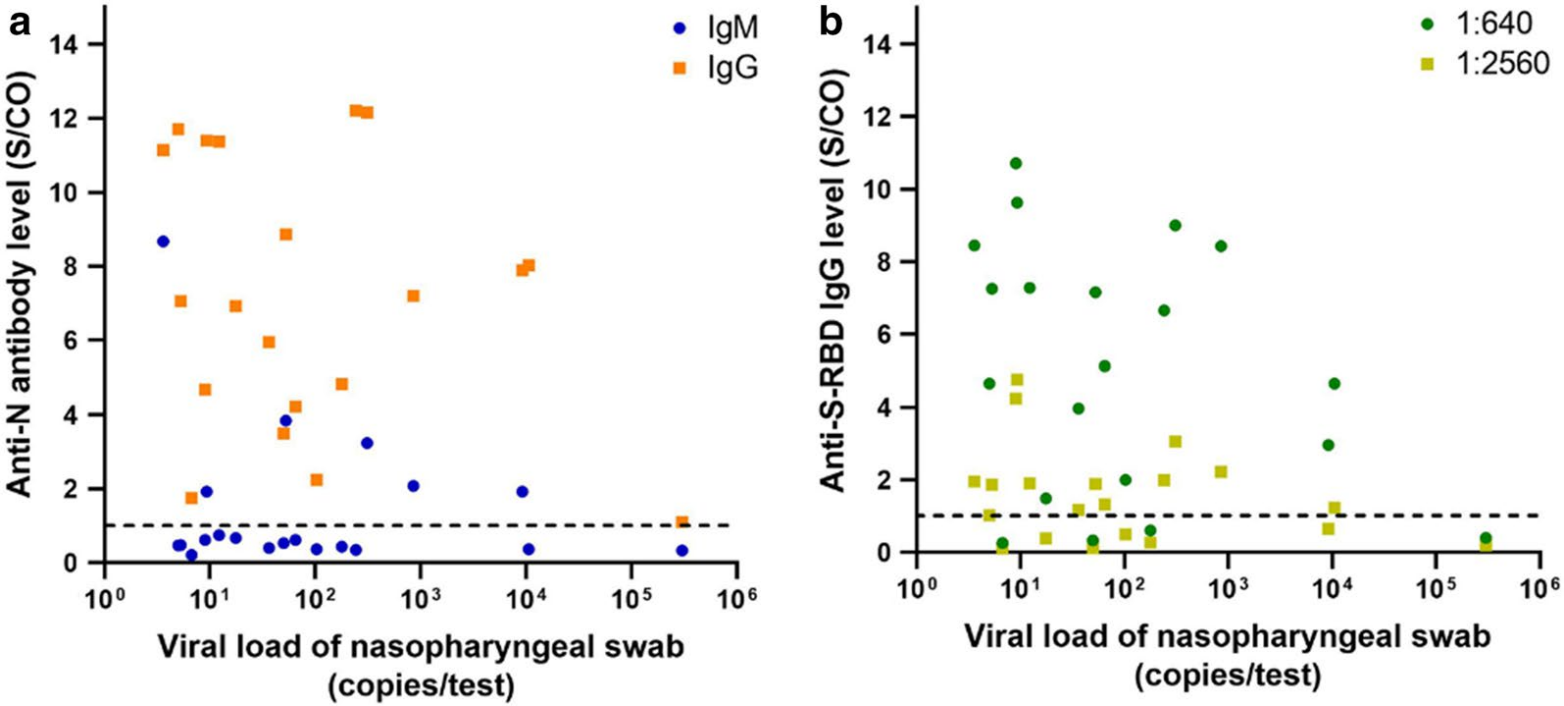
Relationship between **a** anti-N IgG/IgM antibody levels or **b** anti-S-RBD IgG level and viral load of the nasopharyngeal swab. The dashed line shows the threshold (S/CO ratio ≥ 1)

Figure 3a demonstrates the relationship between anti-N IgM/IgG and viral load of the nasopharyngeal swab. For specimens with anti-N IgM levels above the threshold (S/CO ratio ≥ 1), there is a tendency for the antibody level to decrease as viral load increased. Whereas the anti-N IgG level did not have a significant correlation with viral load, and the IgG level was extremely low in a patient with a viral load greater than 10^5^ copies/test. Figure 3b shows the anti-S-RBD IgG level in relation to nasopharyngeal swab viral load, and again, no significant correlation was observed between the anti-S-RBD IgG level and viral load.

## Discussion

This study collected multiple types of specimens from COVID-19 patients with a disease course of 7–69 days, including nasopharyngeal swabs, anal swabs, saliva, blood, and urine specimens. ddPCR was used for nucleic acid detection and absolute quantification of these specimens. ELISA was used to detect the anti-N IgM/IgG and the anti-S-RBD IgG in the serum samples of these patients.

The results of nucleic acid testing of specimens showed that the positive detection rate of nasopharyngeal swabs was the highest, and the average viral load of nasopharyngeal swabs was also the highest. This finding is consistent with the results of existing reports [4]. Research has pointed out that viral infection may damage the gastrointestinal tract, so in the later stages of the disease, the virus may be detected in anal swabs [12], and anal swabs/fecal specimens may remain positive after the nasopharyngeal swabs become negative [13, 14]. The present study also observed this phenomenon, and the positive detection rate of anal swabs was relatively high, reaching 24%. A previous study has shown that the positive detection rate of saliva specimens is high, up to 61.5% [15]. The study found that viral nucleic acids could be detected in posterior oropharyngeal saliva specimens, and a better positive percent agreement was observed in specimens obtained within 7 days after symptoms onset. However, the positive detection rate of saliva specimens in our study was only 16%, which may be related to the sampling method and the day of specimen collection [16]. In summary, our results showed that the nasopharyngeal site is the best for the detection of SARS-CoV-2. Meanwhile, the results also suggested that nucleic acid testing for convalescent patients should be done in nasopharyngeal swabs plus other specimens to get a more accurate diagnosis of full recovery from coronavirus infection since other specimens from patients with negative nasopharyngeal test result were tested positive in this study. It is recommended to test anal swab, because in this study, except for nasopharyngeal swabs, SARS-CoV-2 was mostly detected in anal swabs. Moreover, several studies have characterized the presence of live virus in feces [17–19], indicating that SARS-CoV-2 may be transmitted by fecal route. Notably, Wang et al. [19] failed to isolate virus in feces specimens collected at later time points of disease onset. Therefore, the infectivity of SARS-CoV-2 in different specimens at different time points needs further investigation to better guide the clinical management of COVID-19 patients.

The test results of the antibody level and titer showed that there was no correlation between anti-N IgM/IgG or anti-S-RBD IgG levels with days after symptoms onset or the viral load of nasopharyngeal swabs, which suggested that the antibody level may also be influenced by other factors. The existing literature pointed out that the median (interquartile range, IQR) seroconversion time of anti-N IgM and IgG is 10 days (7–14 days) while the median (IQR) seroconversion time of anti-S-RBD IgG is 13 days (9–17 days). The duration of IgM is short as the IgM level attenuates after reaching the peak at 14 days (9–23 days) while the duration of IgG is longer than IgM [20]. This could explain the results of the present study: most of the patients’ course of disease was more than 2 weeks, so the level of anti-N IgM was low, while anti-N IgG and anti-S-RBD IgG were at a high level.

This study has some limitations. First, the sample size of this study is small, which may cause some correlations fail to be detected. Second, most of the patients in this study had moderate cases, with only four severe or critical patients, so no further multi-parameter analyses could be conducted. Third, the different specimens from each patient were only sampled once and tested once, which may have caused false results due to some pre-analytical and analytical errors.

## Conclusion

COVID-19 is a new infectious disease, and a better understanding of it can help us better prevent and treat the disease. This study analyzed the nucleic acid positive detection rate and viral load of different types of specimens from COVID-19 patients and studied the relationship of antibody levels with days after symptoms onset and viral load. The findings of this study provided the scientific basis for understanding the antibody responses and improving viral nucleic acid sampling and detection.

## Data Availability

All data generated or analyzed during this study are included in this article.

## Abbreviations

SARS-CoV-2: Severe acute respiratory syndrome coronavirus 2
ARDS: Acute respiratory distress syndrome
WHO: World Health Organization
COVID-19: Coronavirus disease 2019
RT-qPCR: Quantitative reverse transcription polymerase chain reaction
ddPCR: Droplet digital polymerase chain reaction
anti-N: Anti-nucleocapsid protein
anti-S-RBD: Anti-spike protein receptor-binding domain
ELISA: Enzyme-linked immunosorbent assay
S: Sample’s OD value
CO: Cut-off value
